# Physicochemical and Biological Characterization of the Proposed Biosimilar Tocilizumab

**DOI:** 10.1155/2017/4926168

**Published:** 2017-03-02

**Authors:** Shiwei Miao, Li Fan, Liang Zhao, Ding Ding, Xiaohui Liu, Haibin Wang, Wen-Song Tan

**Affiliations:** ^1^State Key Laboratory of Bioreactor Engineering, East China University of Science and Technology, Shanghai 200237, China; ^2^Hisun Pharmaceutical (Hangzhou) Co., Ltd., Fuyang, Hangzhou, Zhejiang 311404, China

## Abstract

HS628 has been developed as a proposed biosimilar product of originator tocilizumab (Actemra®). An extensive physicochemical and biological characterization was conducted to assess similarity between HS628 and originator tocilizumab. The amino acid sequence was shown to be identical between HS628 and originator tocilizumab. The higher order structure was found to be indistinguishable from originator tocilizumab. Concerning purity and heterogeneity, HS628 was demonstrated to have similar posttranslational modifications, charge heterogeneity, size heterogeneity, and glycosylation to originator tocilizumab. Moreover, HS628 exhibited highly similar binding affinity and antiproliferative activity as well as capability of inhibiting STAT3 phosphorylation compared to originator tocilizumab. Taken together, HS628 can be considered as a highly similar molecule to originator tocilizumab in terms of physicochemical and biological properties.

## 1. Introduction

Tocilizumab (Actemra) is a recombinant humanized IgG1 monoclonal antibody, which binds to human interleukin-6 (IL-6) receptors (IL-6R). By inhibiting IL-6 binding to both soluble and membrane-bound IL-6R (sIL-6R and mIL-6R), tocilizumab blocks IL-6-mediated signal transduction [1]. Actemra is approved by the US Food and Drug Administration (FDA) for the treatment of rheumatoid arthritis and juvenile idiopathic arthritis patients. It has also been demonstrated that tocilizumab has anticancer potency via apoptosis induction as an agonistic IL-6R regulator and may be utilized as a new target molecule for non-small-cell lung cancer (NSCLC) [2].

According to the FDA, the European Medicines Agency (EMA), and the World Health Organization (WHO) guidelines, biosimilar products must demonstrate similarity in terms of quality, safety, and efficacy with the reference product [3–5]. On March 6, 2015, the FDA approved Sandoz Inc.'s (Sandoz) Zarxio (filgrastim-sndz), as the first biosimilar product for use in the USA. The EMA has already approved a number of biosimilar products, mainly cytokines. CT-P13 (Remsima™; Inflectra™), a biosimilar product of reference infliximab (Remicade®), is the first biosimilar monoclonal antibody approved by the EMA for use in all indications for which Remicade is approved [6].

In 2015, six of the ten top-selling drugs are antibody-based therapeutics. With the increasing use of therapeutic monoclonal antibodies (mAbs), there has been a huge demand for the development of biosimilar mAbs. Regulatory approval for a biosimilar product relies on the demonstration of comparability towards the reference product, starting with an extensive physicochemical and biological characterization, which will provide evidence to support the extent of additional clinical evaluation [7, 8].

A biosimilar product will not be exactly like its reference product, but the critical quality attributes (CQAs) need to match so that the biosimilar product can have similar efficacy, safety, and immunogenicity to those of the reference product [9–11]. A CQA is defined in the ICH Guideline Q8 [12] as a physical, chemical, biological, or microbiological property or characteristic that should be within an appropriate limit, range, or distribution to ensure the desired product quality, safety, and efficacy. Potential CQAs of mAbs may include charge-related variants, size-related variants, oxidation-related variants, glycosylation, structural variants, process-related impurities, and biological properties [13–15]. In the case of adalimumab, the most critical assays are TNF-*α* binding and neutralization, those that directly measured the primary mechanism of action (MOA) of the product [16].

So far, many biosimilar mAbs papers have been published on top-selling mAbs such as adalimumab, rituximab, infliximab, bevacizumab, and trastuzumab [17–21], but not yet on tocilizumab. HS628 has been developed by Hisun Pharmaceutical in China, as a proposed biosimilar tocilizumab of originator tocilizumab. In this study, we describe a subset of the state-of-the-art methods of physicochemical and biological analysis that were performed to demonstrate similarity between HS628 and originator tocilizumab.

## 2. Materials and Methods

### 2.1. Materials

Originator tocilizumab (Actemra 4 mL : 80 mg, 10 mL : 200 mg, and 20 mL : 400 mg) batches were purchased from Roche Pharma (Schweiz) Ltd. (manufactured by Chugai Pharma Manufacturing Co., Ltd.). The proposed biosimilar product, HS628, was produced by Zhejiang Hisun Pharmaceutical (Zhejiang, China). HS628 drug product (4 mL : 80 mg) was used for physicochemical and functional comparability study.

### 2.2. Methods

An array of state-of-the-art and orthogonal analytical techniques were used to compare HS628 with tocilizumab. All chromatographic analyses were performed on Agilent 1260 high performance liquid chromatography (HPLC) systems (Agilent Technologies, Böblingen, Germany). Primary sequences, verified with tryptic peptide mapping and the whole molecule exact masses, were analyzed using reverse-phase ultraperformance liquid chromatography system coupled with a UV detector and a quadrupole time-of-flight mass spectrometer (RP-UPLC-Q-TOF). Higher order structure was evaluated by circular dichroism (CD) and differential scanning calorimetry (DSC). Free proteinogenic thiol group was quantified using Ellman's assay. The disulfide bridging pattern was assessed using RP-UPLC-Q-TOF system after peptide mapping under nonreducing conditions. Posttranslational modifications were identified by RP-UPLC-Q-TOF system after tryptic peptide mapping under reducing conditions. Charge heterogeneity of protein sample with or without carboxypeptidase digestion was assessed by cation exchange chromatography (CEX-HPLC) and imaged capillary isoelectric focusing (icIEF). Size heterogeneity (purity) was determined by size exclusion chromatography (SEC) and capillary electrophoresis-sodium dodecylsulfate (CE-SDS). N-Glycosylation patterns of the products were assessed by LC-MS peptide mapping and normal-phase HPLC with fluorescence detection (NP-HPLC-FL). Functional properties were evaluated by surface plasmon resonance (SPR), antiproliferative assay, and inhibition of STAT3 phosphorylation.

#### 2.2.1. Nonreduced and Reduced Peptide Mapping with Reverse-Phase (RP) HPLC with UV and Mass Spectrometric Detection

Reduced peptide mapping was performed to identify the primary sequences of tested products. Nonreduced peptide mapping was carried out to determine the disulfide bridging pattern. Protein sample was diluted in water at 2 mg/mL and 400 *μ*L of sample solution was mixed with guanidine-HCl in Tris buffer (0.1 mol/L Tris, pH 8.0) to a final concentration of guanidine-HCl of 6 mol/L. The solution was incubated at 37°C for 2 hrs. After incubation, sample solution was reduced with dithiothreitol (DTT) at 10 mg/mL and incubated at 37°C for 30 min. Alkylation was conducted by adding a 20 *μ*L aliquot of a 1 mol/L iodoacetamide stock solution at room temperature for 40 min in the dark. The buffer was exchanged to 0.05 mol/L ammonium bicarbonate at pH 7.8 by ultrafiltration. The protein sample was then digested with sequencing grade trypsin at a 1 : 40 enzyme-sample ratio at 37°C overnight. The enzymatic reaction was quenched with the addition of formic acid to a final concentration of 0.1%. For disulfide bridge identification, the sample was prepared with the same methods as described above without reduction and digested using both trypsin and Glu-C enzyme at a 1 : 1 : 40 ratio of trypsin : Glu-C : sample. RP-UPLC-MS was performed on a Waters ACQUITY UPLC H-Class Bio system coupled with a UV detection and a Q-TOF mass spectrometer. Mobile phase A was water with 0.1% TFA and mobile phase B was acetonitrile with 0.1% TFA. Peptide mapping was conducted using an ACQUITY UPLC BEH 300 C18 column (1.7 *μ*m, 2.1 mm × 150 mm, Waters, USA) with a flow rate of 0.2 mL/min. The gradient started at 5% phase B for 5 min and then increased from 5% phase B to 45% phase B in 45 min, followed by a gradient from 45% phase B to 95% phase B in 10 min, followed by holding at 95% phase B for another 10 min as an elution step. The UV detection wavelength was set at 214 nm. The mass spectrometer was set to run in positive ion mode with a sample cone voltage of 60 V, capillary voltage of 3000 V, mass resolution of 30000, and* m/z* range of 50–2000. The mass spectrometer was calibrated with NaI (2 *μ*g/*μ*L) in water/isopropanol (50/50) and tuned with leucine enkephalin solution (LE, 1 ng/*μ*L) in water/acetonitrile (50/50) with 0.1% formic acid. The ion of LE at* m/z* 556.2771 was used to conduct mass correction. The acquired raw spectra data were then deconvoluted and analyzed using BiopharmaLynx software (version 1.3.3 Build 7).

#### 2.2.2. Molecular Mass Determination Using RP-UPLC-Q-TOF

Sample solution was diluted to a final concentration of 1 mg/mL in 0.05 M Tris-HCl (pH 7.5). Analysis of intact protein and reduced protein was run on a MassPrep™ micro desalting column (Waters, USA) with a flow rate of 0.2 mL/min. The gradient began at 10% phase B for 1 min, and a linear gradient from 10% phase B to 60% phase B in 10 min was then performed, followed by an increase to 90% phase B in 2 min, followed by holding at 90% for another 5 min. The Q-TOF mass spectrometer was run in positive ion mode. Sample cone voltage, capillary voltage, mass resolution, and* m/z* range were set at 60 V, 3000 V, 30000, and 250–4000, respectively. The mass spectrometer was calibrated with NaI solution at a final concentration of 2 *μ*g/*μ*L in water/isopropanol (50/50) and was tuned with leucine enkephalin solution (LE, 1 ng/*μ*L) in water/acetonitrile (50/50) with 0.1% formic acid. The mass correction was conducted using ion of LE at* m/z* 556.2771. The raw spectra data acquired were then deconvoluted and analyzed using BiopharmaLynx software (version 1.3.3 Build 7).

#### 2.2.3. Ellman's Assay

Free proteinogenic thiol group was quantified by using Ellman's reagent (DTNB). L-Cysteine solution (10 mM) was formulated as 100 *μ*M, 75 *μ*M, 50 *μ*M, 25 *μ*M, 20 *μ*M, 10 *μ*M, and 5 *μ*M standard solutions with the assay buffer (100 mM PBS, 1 mM EDTA, pH 7.4). 150 *μ*L 8 M guanidine hydrochloride was added to each well of the microplate. Then, 25 *μ*L test solution or standard solution was added to each well and for each solution 3 replicates were applied. Finally, 2.5 *μ*L Ellman's solution (4 mg/mL 5,5′-dithiobis(2-nitrobenzoic acid)) was added to each well. The absorbance was measured at 412 nm after 3 hrs of incubation. The content of free thiol was determined by external standard method. This method is applicable to detect denatured sample.

#### 2.2.4. Circular Dichroism (CD)

CD experiments were performed on a Chirascan-Plus spectrometer (Applied Photophysics, UK). Near-UV CD spectra were recorded from 250 to 320 nm, wavelength step size was 1 nm, bandwidth was 1 nm, and time per point was 1.5 s. Far-UV CD spectra were recorded within the range of 190–260 nm, wavelength step size was 1 nm, bandwidth was 0.7 nm, and time per point was 0.7 s. For each sample, three scans were performed, and baseline correction was applied. Noise reduction and normalization of the spectrum were performed.

#### 2.2.5. Differential Scanning Calorimetry (DSC)

DSC measurements were carried out on a MicroCal VP-Capillary DSC System (GE Healthcare, USA). Scans were recorded at a rate of 180°C/h. Samples were diluted to 0.5 mg/mL with reference buffer and scanned from 15 to 110°C. For each sample, three scans were performed.

#### 2.2.6. Cation Exchange Chromatography (CEX)

CEX separation was performed using a weak CEX column (ProPac WCX-10, 4 × 250 mm, Dionex, Germany) connected to an Agilent 1260 HPLC system. Mobile phase A consisted of 10 mM sodium phosphate (pH 7.5), and mobile phase B consisted of 10 mM sodium phosphate and 100 mM sodium chloride (pH 7.5). Samples were diluted using equilibration buffer consisting of mobile phases A and B at a ratio of 85 : 15 (v/v) to a final concentration of 2 mg/mL. For C-terminal lysine cleavage, 15 *μ*L of 1 mg/mL carboxypeptidase B (CPB; Roche, Germany) was added to 1 mL of the sample solution, and the mixture was incubated at 37°C for 30 min. The separation gradient was set as follows: 0 to 3 min, holding at 15% B, 3 to 6 min, 15% B–30% B, 6 to 36 min, 30%–70% B, and 36 to 38 min, 70%–100% B. The column was then washed with 100% B for 5 min, followed by equilibration using 15% B for 15 min. The column temperature was set at 30°C and the absorbance of the eluent was monitored at 214 nm.

#### 2.2.7. Imaged Capillary Isoelectric Focusing (icIEF)

Isoelectric point and charge heterogeneity were determined using iCE3 Capillary IEF System (ProteinSimple, USA). The sample was diluted using 8 M urea to a final concentration of 2 mg/mL. 200 *μ*L of sample mixture for icIEF analysis consisted of 70 *μ*L 1% methyl cellulose, 10 *μ*L Pharmalyte (pI 3–10), 0.5 *μ*L pI marker (pI = 7.10), 0.5 *μ*L pI marker (pI = 10.10), 50 *μ*L sample solution in 8 M urea, 50 *μ*L 8 M urea, and 19 *μ*L water. The sample mixture was injected into the cartridge and focused by applying a potential of 1500 V for 1 min for the first focusing step and a potential of 3000 V for 10 min as the second focusing step. The isoelectric point (pI) and charge variants were analyzed using Chrom Perfect Analysis software.

#### 2.2.8. Size Exclusion Chromatography (SEC)

SEC was conducted on a TSK-Gel G3000SW_XL_ column (Tosoh Bioscience, Japan) connected to an Agilent 1260 HPLC system. The SEC separation buffer contained 20 mM PBS and 200 mM sodium chloride at pH 7.5. 20 *μ*L of protein sample at a concentration of 2 mg/mL was injected onto the column. The separation was conducted at a flow rate of 0.5 mL/min and was monitored at 280 nm. Molecular weight determination of monomers and polymers was tested by SEC-MALS-RI. Multiangle static Light Scattering (MALS) detector was DAWN HELEOS II (Wyatt Technology, USA), and Refractive Index (RI) detector was Optilab T-rEX (Wyatt Technology, USA).

#### 2.2.9. Capillary Electrophoresis with Sodium Dodecyl Sulfate (CE-SDS)

CE-SDS was performed on 7100 CE System (Agilent, USA) equipped with a diode array detector. For nonreduced CE-SDS analysis, the antibody samples at a final concentration of 1 mg/mL were mixed with SDS sample buffer (10% v/v) and 12.5 mM iodoacetamide. The mixture was heated at 70°C in a water bath for 10 min, cooled at room temperature, and centrifuged at 10000 rpm for 5 min for injection. For reduced CE-SDS, the samples were treated as described above with *β*-mercaptoethanol (5% v/v) instead of iodoacetamide. Before separation, the capillary was rinsed with 0.1 mol/L NaOH for 2 min and 0.1 mol/L HCl for 1 min, followed by water for 1 min. Then, the capillary was filled with SDS sieving gel buffer for another 15 min in forward direction. The samples were injected at the anode at −10 kV for 30 s and separated at −15 kV with reverse polarity for 40 min.

#### 2.2.10. Glycan Analysis

The N-glycan analysis was carried out using normal-phase HPLC florescence (NP-HPLC-FL) approach. The N-linked oligosaccharides of protein were released by incubation with PNGase F. 0.2 mg protein was mixed with 0.1 U PNGase F in 20 mM Tris-HCl buffer (pH 7.4) and incubated at 37°C overnight. The deglycosylated protein was then precipitated by adding precooled ethanol and incubated at −20°C for a further 2 hrs. The mixture containing released N-glycans was centrifuged at 10000 rpm at 4°C for 0.5 hrs and the supernatant was completely dried using vacuum centrifugation. Dried N-glycan was mixed with 20 *μ*L labeling reagent (0.4 mol/L 2-aminobenzamide, 1 mol/L NaCNBH_3_ in 3 : 7 (v : v) acetic acid : DMSO) and incubated at 65°C for 4 hrs in the dark. The mixture was then cleaned using Ludger Clean S-cartridges (Ludger, UK) according to the manufacturers' instructions. The labeled N-glycans were separated on an ACQUITY UPLC BEH Glycan column at 0.2 mL/min (1.7 *μ*m, 2.1 × 150 mm, Waters) linked to an Agilent 1290 UPLC system with fluorescence detector (excitation at 330 nm, emission at 420 nm). Mobile phase A was 125 mmol/L ammonium formate (pH 4.4) in water and mobile phase B was 100% acetonitrile. The separation began with 20% phase A from 0 to 5 min, and a linear gradient of mobile phase A from 20% to 45% was performed to separate the labeled N-glycans, followed by an elution step with 60% mobile phase A for a further 15 min.

#### 2.2.11. Surface Plasmon Resonance (SPR) Binding

The kinetic interactions of protein samples with IL-6R, Fc*γ*RIIIa, and FcRn were evaluated using a Biacore X100 plus biosensor. For IL-6R affinity analysis, anti-Fc antibody was immobilized on a CM5 sensor chip surface according to the manufacturer's recommendation with a level of ~2500 response units (RU) reached. The protein sample at a concentration of 1.0 *μ*g/mL was injected at a flow rate of 10 *μ*L/min for 60 s and captured by the anti-Fc antibody. IL-6R in a series of concentrations ranging from 150 ng/mL to 4.7 ng/mL was injected into the flow cells at a flow rate of 30 *μ*L/min for 180 s, with a dissociation time of 500 s using HBS-EP+ buffer. For Fc*γ*RIIIa and FcRn binding determination, anti-His antibody was immobilized on a CM5 sensor chip surface as recommended by the manufacturer's instructions with an RU of ~2500. The protein sample at 1.0 *μ*g/mL was injected into the flow cells at 10 *μ*L/min for 60 s and captured by the anti-His antibody. Fc*γ*RIIIa and FcRn in serially diluted concentrations from 150 ng/mL to 4.7 ng/mL were injected at a flow rate of 30 *μ*L/min for 180 s. The dissociation phase using HBS-EP+ buffer was then set to 500 s. The primary sensorgram data was processed using Biacore evaluation software 1.0 for the determination of association rate constant (*k*_*a*_), dissociation rate constant (*k*_*d*_), and dissociation equilibrium constant (*K*_*D*_) data.

#### 2.2.12. Antiproliferation Assay

DS-1 cells (ATCC® CRL-11102™) were seeded in RPMI-1640 media with 10% FBS and incubated in 96-well plates at 37°C. A series of concentrations ranging from 800 *μ*g/mL to 12.21 ng/mL of HS628 and originator tocilizumab were added and incubated for 72 hrs. CCK-8 (Dojindo, Japan) was added to stain the cells and incubated at 37°C for 3 hrs. The absorbance was measured at 450 nm and 650 nm. Test results were expressed as the relative percentage of the EC50 from the dose-response curve of HS628 with respect to originator tocilizumab.

#### 2.2.13. Inhibition of STAT3 Phosphorylation

U937 cells (ATCC CRL-1593.2™) in the logarithmic growth phase were starved in RPMI-1640 media, with HS628, originator tocilizumab, and an irrelevant antibody control (anti-CD20 mab) at 37°C for 3 hrs. The cells were pulsed or not with rhu-IL6 (Prospec, Israel) for 15 min. The cells were then fixed (BD Cytofix Fixation Buffer, BD Biosciences, USA) for 12 min, permeabilized (Perm Buffer III, BD Biosciences) for 30 min, and incubated with Alexa Fluor® 647 Mouse Anti-Stat3 (BD Biosciences) in BD Pharmingen Stain Buffer for 1 hour at room temperature. Cells were analyzed on BD FACSCalibur instrument (BD, USA).

## 3. Results and Discussion

HS628, a biosimilar product of originator tocilizumab, was produced in a genetically engineered Chinese hamster ovary (CHO) cell line. The gene sequences of HS628 were reverse-engineered from the amino acid sequence of originator tocilizumab. A company-internal selection of expression vectors and transfection and selection methods were used for cell line development. The upstream process was performed in fed-batch mode in a bioreactor with a proprietary chemically defined medium. Feeding of various nutrients including galactose appeared to be very critical to maintain the glycan profiling of HS628 as similar as possible to that of originator tocilizumab. Following upstream production, HS628 was purified by industry standard chromatographic purification and viral inactivation methods, which consist of Protein A affinity, anion exchange chromatography, and cation exchange chromatography, in combination with viral inactivation (low pH), virus clearance (nanofiltration), and ultrafiltration/diafiltration (UF/DF) steps. Downstream process efficiency was established adequately for the removal of process-related impurities (e.g., host cell protein and DNA) and product-related impurities (e.g., aggregates). The residual levels of process-related impurities comply with existing guidelines for biopharmaceuticals. The capacity of viral inactivation and clearance procedures have been validated by National Institutes for Food and Drug Control (China). The drug substance was formulated to the final drug product using the same formulation as the originator tocilizumab.

A candidate biosimilar product should be similar to the originator product in terms of physicochemical characteristics and functional properties [22, 23]. To demonstrate the biosimilarity, the state-of-the-art robust methodologies (Table 1) including chromatographic, electrophoretic, and cell-based bioassay methods, along with CD, DSC, MS, and SPR technologies, were employed to compare biosimilar tocilizumab HS628 and originator tocilizumab. The selected methods are widely used in similarity assessment of biosimilar products, which are capable of detecting minor differences in the protein structures, as well as identifying and quantifying product-related variants [24–26].

### 3.1. Physicochemical Properties

**Figure 1:**
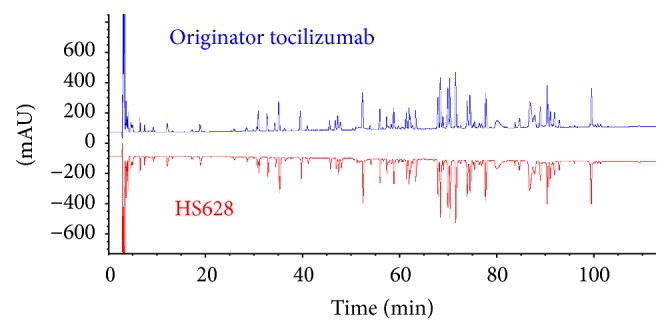
Mirror plot of peptide mapping chromatograms obtained from RP-UPLC-UV for originator tocilizumab and HS628.

**Figure 2:**
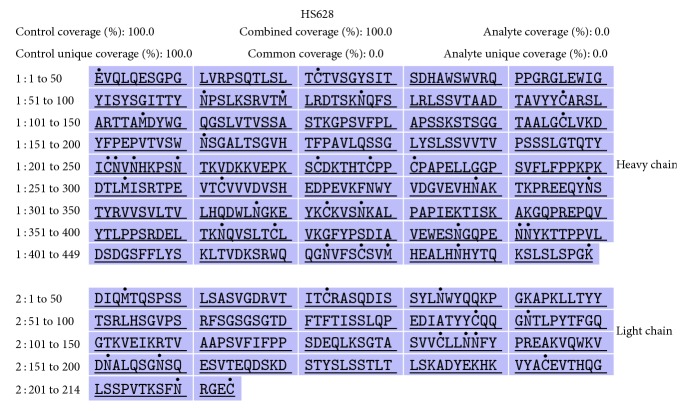
Sequence coverage of the heavy chain and light chain of HS628.

**Figure 3:**
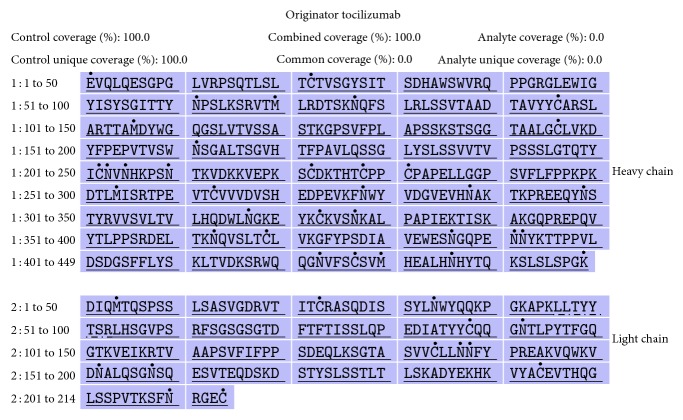
Sequence coverage of the heavy chain and light chain of originator tocilizumab.

*Primary Structure*. The RP-HPLC-UV tryptic peptide map of the biosimilar HS628 showed indistinguishable chromatograms of the fragmented peptides from the originator tocilizumab (Figure 1). Primary amino acid sequence analysis of the peptide fragments of HS628 and originator tocilizumab was obtained by using reduced peptide mapping followed by RP-UPLC-MS sequencing, which resulted in 100% sequence coverage for the heavy chain and light chain of two products. (Figures 2 and 3). The sequences obtained of HS628 were found to be identical to the sequences obtained with originator tocilizumab, which were matched with the theoretical sequence of Actemra [27]. Mass analysis of intact and reduced (heavy chain and light chain) forms of HS628 by RP-UPLC-MS was observed to show consistent molecular masses with those of the originator tocilizumab (Table 2).

*Higher Order Structure*. Nonreducing peptide maps showed the expected disulfide bridging pattern in both originator product and HS628. The following disulfide bridges were identified: intrachain [Cys(L23)-Cys(L88), Cys(L134)-Cys(L194), Cys(H22)-Cys(H96), Cys (H146)-Cys(H202), Cys(H263)-Cys(H323), Cys(H369)-Cys(H427)] and interchain [Cys(L214)-Cys(H222), Cys(H228)-Cys(H228), Cys(H231)-Cys(H231)]. The results of Ellman's assay showed that the number of moles of free SH groups per mole of IgG was comparable in HS628 and originator tocilizumab and in the range of 0.4 (free SH mol/IgG mol).

**Figure 4:**
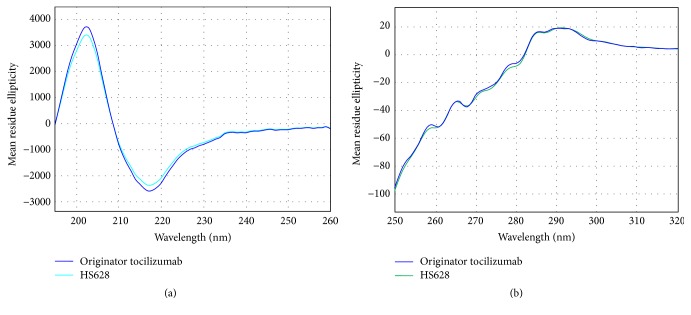
Comparison of CD spectra of HS628 and originator tocilizumab. (a) Far-UV spectra, (b) near-UV spectra.

The secondary structure (*α*-helix, *β*-sheet, and random coil) and tertiary structure can be determined by CD spectroscopy in the far-UV region and near-UV region, respectively. Far-UV CD spectra (Figure 4(a)) and near-UV CD spectra (Figure 4(b)) of HS628 and originator tocilizumab were shown to overlap with each other, and the contents of *α*-helix, *β*-sheet, and random coil of the two products were comparable, respectively.

**Figure 5:**
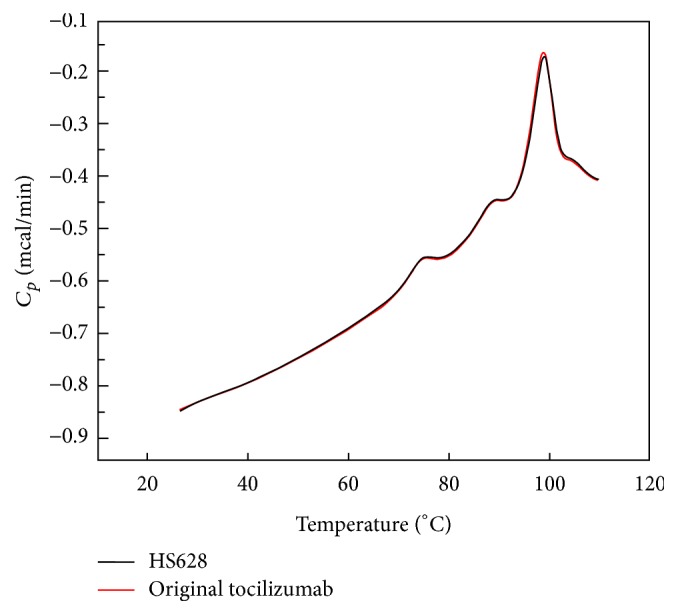
Comparison of DSC spectra of HS628 and originator tocilizumab.

The thermodynamic stability of HS628 and originator tocilizumab was evaluated by differential scanning calorimetry (DSC). The DSC thermograms of HS628 and originator tocilizumab were found to be superimposable. Transition temperatures (*T*_*m*_) of HS628 by DSC (Figure 5) were 74.4°C, 88.6°C, and 98.7°C, whereas, for the originator tocilizumab, they were 74.4°C, 88.6°C, and 98.6°C. The three *T*_*m*_ values are considered to be linked to the unfolding of the CH2, Fab, and CH3 domain, respectively [28].

Collectively, disulfide bridging pattern, free thiol content, CD, and DSC analyses confirmed that the higher order structure of HS628 was comparable to that of originator tocilizumab.

*Posttranslational Modifications*. RP-HPLC-UV/MS peptide mapping was used to detect posttranslational modifications, such as deamidation, oxidation, and phosphorylation. Similarity levels of deamidation and oxidation modifications were detected in HS628 and originator tocilizumab (Table 3). Both the number and the locations of these modifications were generally consistent between HS628 and originator tocilizumab.

Deamidation of the heavy chain and light chain of HS628 and originator tocilizumab was observed at the positions HC-Asn^77^, HC-Asn^288^, HC-Asn^317^, HC-Asn^386^, HC-Asn^436^, LC-Asn^34^, LC-Asn^137^, and LC-Asn^138^, and the levels of deamidation of the two products were comparable.

No detectable Met oxidation was observed on the light chain of the two products. Similar low levels of Met oxidation on the heavy chain of HS628 and originator tocilizumab were observed at the position of HC-Met254.

No detectable phosphorylation was observed in HS628 or originator tocilizumab.

*Charge Heterogeneity*. Antibody charge variants can be formed by chemical and enzymatic modifications [29].

**Figure 6:**
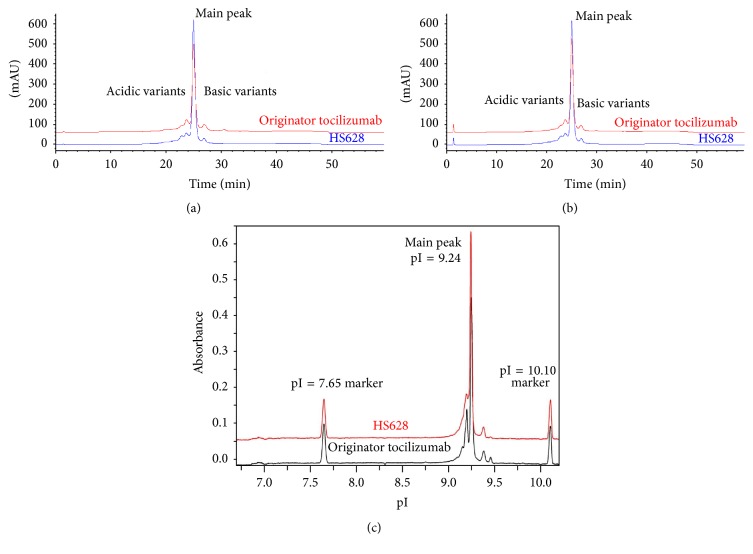
Charge heterogeneity comparison of HS628 and originator tocilizumab. (a) CEX chromatograms of HS628 and originator tocilizumab, (b) CEX chromatograms of HS628 and originator tocilizumab after CPB digest, and (c) cIEF chromatograms of HS628 and originator tocilizumab.

Charge heterogeneity may substantially affect stability, biological activity, and pharmacokinetics of antibodies [30]. Cation exchange chromatography (CEX) was used to assess the profile of charge variants that may be more acidic or basic relative to the main peak. It was revealed that the average abundances of the main peak, acidic variants, and basic variants were within the same order of magnitude for HS628 and originator tocilizumab (Figure 6(a)), with the mean values of 68.8%, 25.2%, and 6.0% (*n* = 4) for HS628 and 63.3%, 25.0%, and 11.7% (*n* = 4) for originator tocilizumab, respectively. The sum of the HS628 acidic variants was comparable to that of originator tocilizumab, while the sum of the basic variants was found to be slightly lower than that of originator tocilizumab. Treatment of the mAbs with CPB removes C-terminal lysine heterogeneity. The results obtained after digestion with CPB (Figure 6(b)) showed also a comparable content of charge variants between the two products, with the average abundances of the main peak, acidic variants, and basic variants of 68.7%, 25.3%, and 6.0% (*n* = 4) for HS628 and 65.8%, 25.7%, and 8.5% (*n* = 4) for originator tocilizumab, respectively.

An orthogonal analytical technique for the evaluation of charge heterogeneity is imaged capillary isoelectric focusing (icIEF). The isoelectric points (pI) for the main peak were 9.24 for both HS628 and originator tocilizumab, and the contents of the main peak of the two products were 66.9% and 64.7%, respectively (Figure 6(c)). Consistent with the CEX results, no significant differences were observed in the cIEF chromatograms of the biosimilar HS628 and originator tocilizumab, indicating the comparable charge heterogeneity between the two products.

*Size Heterogeneity*. It is known that protein aggregates in immunomodulatory biologic formulations can trigger an unwanted immunogenic response [31–33]. Size exclusion chromatography (SEC) was performed to detect the levels of aggregates, monomers, and fragments. The retention times of monomeric HS628 and originator tocilizumab were the same, while average purity was 99.4% for HS628 (*n* = 4) and 99.0% for originator tocilizumab (*n* = 4). No fragments (low molecular weight variants) were found in both HS628 and originator tocilizumab, and there was also no indication of different types of high molecular weight variants. SEC-MALS-RI results showed that the molecular weights of the monomer and the dimer were 152 KDa and 301 KDa, respectively.

**Figure 7:**
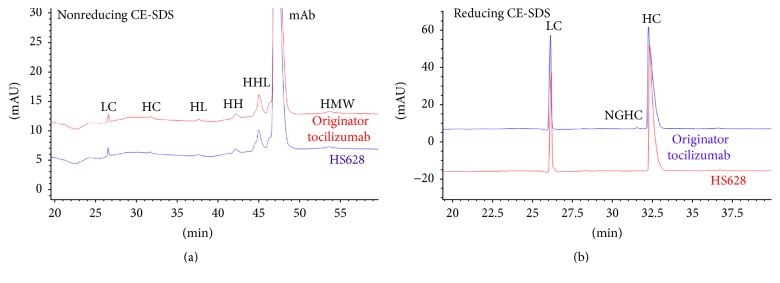
Comparison of CE-SDS electropherograms between HS628 and originator tocilizumab. (a) Nonreducing, (b) reducing. LC: light chain; HC: heavy chain; HL: heavy-light chain; HH: heavy-heavy chain; HHL: heavy-heavy-light chain; HMW: high molecular weight substance; NGHC: nonglycosylated heavy chain.

Nonreducing CE-SDS was used to separate monomers from higher molecular weight variants and fragments [HHL (125 kDa), HH (100 kDa), HL (75 kDa), HC (50 kDa), and LC (25 kDa)]. In the electropherograms of HS628 and originator tocilizumab, a number of fragments were resolved and detected. The amounts of free light chain (LC), free heavy chain (HC), HL, HH, and HHL of HS628 and originator tocilizumab were comparable, and the average percentage of mAb (monomer) was 95.3% for HS628 (*n* = 4) and 95.0% for originator tocilizumab (*n* = 4) (Figure 7(a)).

Reducing CE-SDS was used to assess the levels of light chain, heavy chain, and nonglycosylated heavy chain (NGHC). Similar chromatographic profiles and purities (HC + LC) of 99.0% were found for HS628 and originator tocilizumab (Figure 7(b)). The average level of NGHC of HS628 was 0.14% (*n* = 4), which was slightly lower than that of originator tocilizumab (0.72%, *n* = 4).

Overall, the results from SEC and CE-SDS show that HS628 has a similar purity and aggregate level to originator tocilizumab.

**Figure 8:**
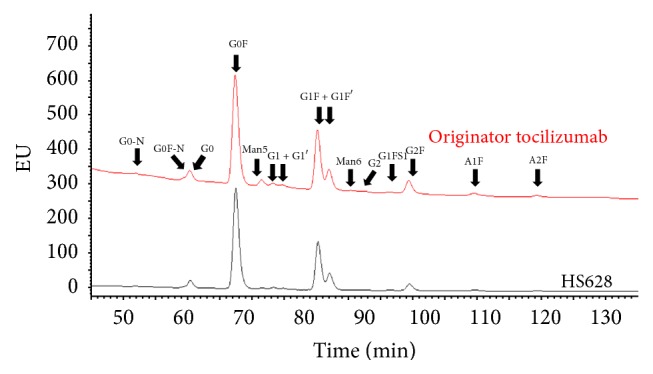
Comparison of the glycosylation pattern of HS628 and originator tocilizumab.

*Glycosylation*. Glycosylation has been identified as a CQA for many antibody-based drugs [34–36]. LC-MS/MS peptide mapping was used to characterize the glycosylation of the two mAbs. Peptide mapping confirmed the glycosylation site of HS628 and originator tocilizumab at Asn299, while reduced CE-SDS revealed that in both molecules >99% of Asn299 was glycosylated. N-linked glycans were released from tocilizumab by enzymatic hydrolysis using PNGaseF and then were labeled with 2-aminobenzamide followed by normal-phase HPLC with fluorescence detection (NP-HPLC-FL). The glycan patterns of HS628 and originator tocilizumab were comprised of the same principal glycoforms (Figure 8). It should be noted here that no potential immunogenic glycoforms such as NGNA residues were observed. The glycosylation pattern of the major abundant glycans, such as the G0, G0F, G1F, G1'F, and G2F isoforms, was very similar between HS628 and originator tocilizumab. Small differences could be identified when looking at the low abundant glycan structures. HS628 was shown to contain slightly lower amounts of mannose structures (Man5, Man6) of around 0.7% than those of the originator (1.5–2.5%).

Together, HS628 and the originator share similar pattern and abundance of various glycan moieties.

### 3.2. Functional Characterization

A comprehensive set of potency bioassays including SPR binding assays, antiproliferation assay, and inhibition of STAT3 phosphorylation were developed to provide a complete evaluation of HS628's functional integrity and comparability to originator tocilizumab.

*SPR Binding Assays*. SPR technique was used to determine the affinity constants of HS628 and originator tocilizumab to sIL-6R, recombinant human Fc*γ*RIIIa, and FcRn receptors in parallel. Fc*γ*RIIIa is implicated in inducing ADCC (antibody-dependent cell-mediated cytotoxicity). Although the mechanism of action of tocilizumab does not involve ADCC, the binding activity of tocilizumab to Fc*γ*RIIIa was also assessed. The results show that the affinities of HS628 towards sIL-6R, Fc*γ*RIIIa, and FcRn were very comparable to those of the originator product, respectively (Table 4); the data shown were performed head to head and reflect the minimum and maximum value of four originator batches and four HS628 batches.

**Figure 9:**
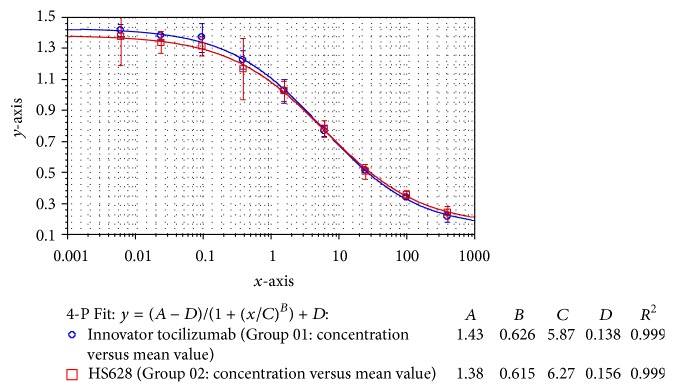
DS-1 cell growth inhibition curves of HS628 and originator tocilizumab.

*Antiproliferation Assay*. In the cell-based bioassay, the cell growth inhibiting activity was evaluated by adding IL-6 and HS628/originator tocilizumab to DS-1 cells such that they compete for the IL-6R on the cells. The results demonstrated that both products have the same potency to deplete DS-1 cells, being the mean relative potencies towards the originator tocilizumab of 98%, 102%, 94%, and 105% for four batches of HS628. One of these curves was shown in Figure 9, indicating that HS628 had a comparable biological potency to originator tocilizumab.

*Inhibition of STAT3 Phosphorylation*. STAT3, a member of STAT family, has been described in mediating IL-6 signaling through interaction with the IL-6R, and IL-6 can induce phosphorylation of STAT3 [37].

**Figure 10:**
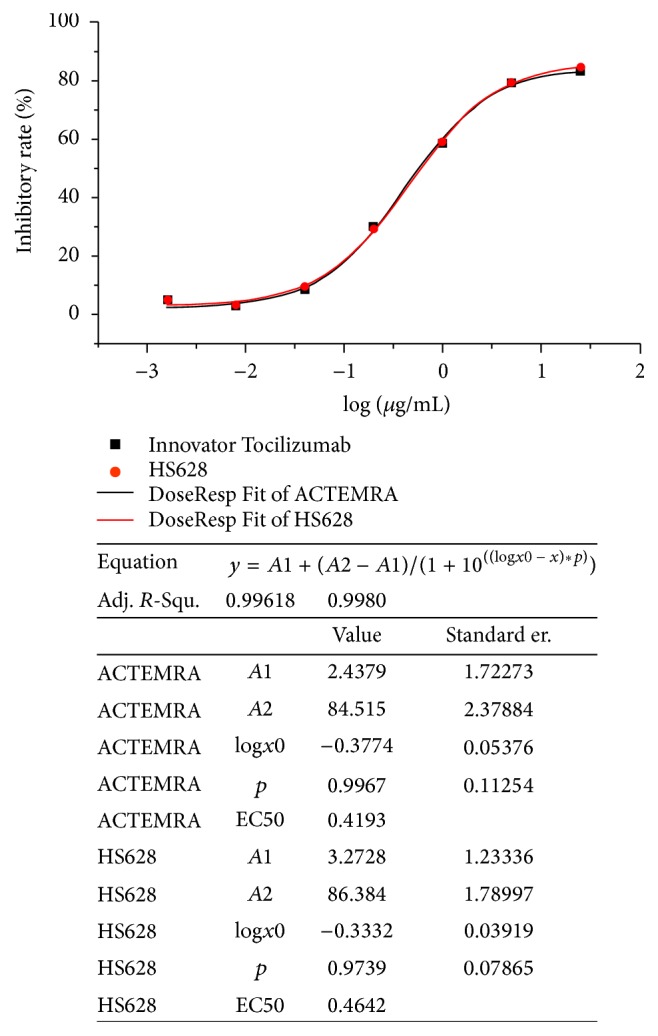
Comparison of inhibition of STAT3 phosphorylation between HS628 and originator tocilizumab (Actemra). The dose-response curves of inhibition effects on STAT3 phosphorylation of HS628 and originator tocilizumab (Actemra) were generated using 4-parameter-curve equation* y* =* A*1 + (*A*2 −* A*1)/(1 + 10^((⁡log*x*0 − *x*)*∗p*)^) by the software OriginPro (Version 8.6.0 Sr2). The EC50 values of STAT3 phosphorylation inhibition of HS628 and originator were calculated from the log*x*0 values from the corresponding curves. The results showed that the dose-response curves of HS628 and tocilizumab were highly overlapped, indicating that the inhibition effect on STAT3 phosphorylation of HS628 was highly comparable with that of originator tocilizumab (Actemra).

Fluorescence Activated Cell Sorting (FACS) was used to assess the biological activity of HS628 and originator tocilizumab to inhibit IL-6-induced STAT3 phosphorylation in U937 cells. An irrelevant antibody control (anti-CD20 mAb) was used as negative control. The results showed that IL-6 could induce 43.02% STAT3 phosphorylation in U937 cells, while the rate of STAT3 phosphorylation was only 0.99% without IL-6. The anti-CD20 mAb did not block IL-6-induced STAT3 phosphorylation since the rate of STAT3 phosphorylation was still 42.66%. Both HS628 and originator tocilizumab could inhibit STAT3 phosphorylation in U937 cells, as shown in Figure 10. An overlapping sigmoidal dose-response curve was obtained for both HS628 and originator tocilizumab. Half-maximal effective concentration (EC50) value calculated for HS628 from the dose-response curve was found to be similar to that of originator tocilizumab.

Functional similarity is a very critical component of the totality of evidence required for demonstration of biosimilarity. These results collectively demonstrated the high level of similarity in functional properties between HS628 and originator tocilizumab.

## 4. Conclusions

In the present work, the comprehensive physicochemical and biological characterization, including the verification of primary structure, higher order structure, posttranslational modifications, charge heterogeneity, size heterogeneity, glycosylation, binding affinity to IL-6R, Fc*γ*RIIIa, and FcRn, antiproliferative activity, and inhibition of STAT3 phosphorylation, provided solid evidence to prove the similarity between the proposed biosimilar HS628 and originator tocilizumab. Based on this research, HS628 can be considered as a highly similar molecule to originator tocilizumab in terms of physicochemical and biological properties.

Subsequently, a comparative animal toxicity study was conducted to evaluate the safety of the product in accordance with Good Laboratory Practice (GLP) (China Food and Drug Administration (CFDA), 2003). No significant differences between HS628 and originator tocilizumab were observed through the comparative toxicological studies in animals. In addition, HS628 was observed to exhibit a similar pharmacokinetics profile compared to that of the originator tocilizumab following a single-dose injection in cynomolgus. As such, it can be anticipated that the proposed biosimilar HS628 would show comparable potency and safety as the reference originator product in future clinical trials.

## Figures and Tables

**Table 1:** Characterization strategy for the proposed biosimilar HS628 and originator tocilizumab.

Category	Attributes	Methods
Primary structure	Amino acid sequence	Reduced RP-UPLC-MS peptide mapping
	Molecular weight	RP-UPLC-MS

Higher order structure	Disulfide bridging	Nonreduced RP-UPLC-MS peptide mapping
	Free thiols	Ellman's assay
	Secondary and tertiary structure	Circular dichroism (CD)
	Thermodynamic stability	Differential scanning calorimetry (DSC)

Posttranslational modifications	Deamidation, oxidation	Reduced RP-UPLC-MS peptide mapping

Charge heterogeneity	Charge variants	CEX-HPLC
	Isoelectric point	Imaged capillary isoelectric focusing (icIEF)

Size heterogeneity	High molecular weight impurities	SEC-HPLC
	Low molecular weight impurities	Nonreduced CE-SDS
	Nonglycosylated heavy chain (NGHC)	Reduced CE-SDS

Glycosylation	Glycan profile	NP-HPLC-FL

Binding activity	Affinity to IL-6R, Fc*γ*RIIIa, and FcRn	Surface plasmon resonance

Potency	Antiproliferative potency	Antiproliferation assay
	Signal transduction inhibition	Inhibition of STAT3 phosphorylation

**Table 2:** The whole molecule, heavy chain, and light chain exact masses of originator tocilizumab and HS628 by MS.

Type		Originator tocilizumab (4 bathes) Mass (Da)				HS628 (4 bathes) Mass (Da)			
		B2014B10	B2019B26	B1018B13	B2027B13	20130901	20130902	20130903	20130904
Whole molecule	G0F/G0F	147885.19	147884.44	147885.81	147885.61	147885.78	147887.00	147882.39	147887.77
	G0F/G1F	148046.00	148045.88	148046.81	148046.61	148046.27	148048.14	148044.20	148048.41
	G1F/G1F	148207.06	148206.64	148207.81	148207.63	148208.34	148208.23	148204.53	148209.59
	G1F/G2F	148365.19	148364.81	148365.66	148365.53	148366.00	148369.11	148364.52	148371.30
	Deglycosylated molecule	144998.63	144996.91	144997.30	144997.67	144997.02	144997.81	144994.66	144999.08

Heavy chain	G0F	50445.32	50445.18	50445.46	50445.50	50443.57	50443.88	50444.11	50443.75
	G1F	50606.94	50606.87	50606.97	50607.01	50605.44	50605.91	50605.71	50605.55
	G2F	50767.95	50767.80	50767.92	50767.96	50772.80	50772.06	50774.06	50776.70
	Deglycosylated molecule	48999.88	48999.73	48999.92	49000.03	48997.97	48998.05	48998.36	48998.13

Light chain		23500.97	23500.96	23500.97	23500.96	23500.75	23500.75	23500.77	23500.76

**Table 3:** Deamidation and oxidation modifications of HS628 and originator tocilizumab.

	Modification site	Modification type	Mass signal intensity (%)				
			HS628 (20130901)	HS628 (20130902)	HS628 (20130903)	HS628 (20130904)	Originator tocilizumab (B2014B10)
Heavy chain	N(77)QFSLR	Deamidation	5.2	4.2	3.8	3.9	3.7
	DTLM(254)ISR	Oxidation	2.5	1.5	1.9	1.8	3.3
	FNWYVDGVEVH N(288)AK	Deamidation	22.6	21.8	20.6	20.5	21.4
	VVSVLTVLHQDWL N(317)GK	Deamidation	99.8	99.7	99.8	99.6	99.7
	GFYPSDIAVEWES N(386)GQPENNYK	Deamidation	75.6	82.6	74.9	76.6	75.5
	WQQGNVFSCSVMHE ALHN(436)HYTQK	Deamidation	6.7	7.7	6.7	6.2	6.8

Light chain	ASQDISSYLN(34)WYQQKPGK	Deamidation	1.9	2.5	1.5	1.9	1.6
	SGTASVVCLL N(137)NFYPR	Deamidation	44.4	37.8	34.2	35.4	41.5
	SGTASVVCLLN N(138)FYPR	Deamidation	5.9	5.5	4.7	4.9	5.8

**Table 4:** Affinity constants (*K*_*D*_) for binding to sIL-6R, Fc*γ*RIIIa, and FcRn receptors as determined by Biacore SPR.

	Originator tocilizumab *K*_*D*_	HS628 *K*_*D*_
sIL-6R	1.08~1.78 nM	0.92~1.17 nM
Fc*γ*RIIIa	0.21~0.38 *μ*M	0.24~0.25 *μ*M
FcRn	0.29~0.31 *μ*M	0.25~0.26 *μ*M
